# Facilitators and barriers to adopting or expanding medications for opioid use disorder provision in rural Colorado jails: a qualitative analysis

**DOI:** 10.1186/s40352-024-00280-x

**Published:** 2024-06-06

**Authors:** Heidi L. McNeely, Terri L. Schreiber, William L. Swann, Claudia R. Amura

**Affiliations:** 1 The Schreiber Research Group, P.O. Box 371342, Denver, CO 80237 USA; 2 School of Public Administration, College of Public Affairs and Community Service, University of Nebraska at Omaha, 6001 Dodge Street, CPACS 111, Omaha, NE USA; 3 College of Nursing, University of Colorado Anschutz Medical Campus, 13120 East 19th Avenue, 3rd Floor - Room 3255, Aurora, CO 80045 USA

**Keywords:** Medications for opioid use disorder, Substance use disorder treatment, Jail-based services, Correctional health

## Abstract

**Background:**

Opioid use disorder (OUD) is common among individuals who are incarcerated. However, OUD treatment services are sparse in smaller county jails found in many rural areas, which limits a healthy and supportive jail environment. This study assesses the facilitators of and barriers to medications for opioid use disorder (MOUD) adoption or expansion in rural Colorado jails. A qualitative descriptive design was implemented during the summer of 2022 using semi-structured interviews with jail staff, sheriffs, and contracted personnel. Interview questions focused on facilitators of existing MOUD services and barriers to adopting or expanding services. To identify the facilitators and barriers, data were coded using thematic analysis.

**Results:**

Seven jails were included in the study. Representatives from each jail participated in the seven interviews, which often included multiple participants per interview. Three of the jails had established routine practices for MOUD administration. Two jails occasionally administered MOUD or had plans in place to be able to administer, while the remaining two did not offer any MOUD. While administrative support, collaborative partnerships, and jail nurses facilitated MOUD use, barriers were more prevalent, including physical space limitations, distance to services, lack of providers in the area, staffing and training issues, funding/budget issues, and perceived risk of diversion.

**Conclusion:**

Making MOUD available to people who are incarcerated is an important and timely step in enhancing the jail environment, especially in rural areas that often lack access to MOUD. As states look to require MOUD availability for people who are incarcerated, facilitators to MOUD adoption/expansion can be leveraged while strategies are needed to overcome barriers.

## Introduction

Opioid use disorder (OUD) is a life-threatening condition associated with a 20-fold greater risk of early death due to overdose, infectious diseases, trauma, and suicide (National Academies of Sciences, Engineering, and Medicine, 2019). In 2022, over 105,000 people died of a drug overdose, and 75% of such deaths were due to opioids (Centers for Disease Control and Prevention, 2023).

Medications for opioid use disorder (MOUD) include three Food and Drug Administration (FDA)-approved drugs (buprenorphine, methadone, and naltrexone) and are considered the gold standard treatment for OUD (Substance Abuse and Mental Health Services Administration, 2023). As drug use and OUD contribute to crime and incarceration (Bennett et al., 2008), timely access to MOUD can promote a healthy and supportive correctional environment and a successful recovery. However, when incarcerated, individuals with illicit opioid use, or use beyond what is prescribed, stop having access. This typically triggers a precipitous withdrawal and makes them especially vulnerable to overdose upon release given their reduced tolerance for the drug during incarceration (Krinsky et al., 2009). These individuals also lose access to public health insurance (Medicaid), which means there is no insurance coverage for services that must be brought in from outside the jail and these services (including medication needs) are billed to the jail directly for the individual to receive. While law enforcement-assisted diversion, jail-based case management for OUD, and referral networks for post-discharge treatment are all viable methods for increasing coordination and access to health services post-release (Hamilton & Belenko, 2016), treatment while incarcerated with enhanced health coordination with the justice system is a pressing need.

With the prevalence of substance use disorder (SUD)/OUD in jails ranging from 27 to 65% (Ferguson et al., 2019), there is a significant population that could benefit from MOUD during incarceration. MOUD has been shown to improve health, reduce craving and withdrawal symptoms, and improve financial and social outcomes for people who are incarcerated (Komalasari et al., 2021). MOUD provided during incarceration also increases engagement in community-based treatment and lowers risks associated with opioid use post-release (Cates & Brown, 2023). Contrarily, those without access to MOUD during incarceration or upon release are at higher risk for recidivism (Evans et al., 2022; Surratt et al., 2018) and overdose death (Binswanger et al., 2007).

Despite these benefits, the adoption of MOUD in US correctional settings has been slow (Brezel et al., 2020; Clarke et al., 2018). Barriers to implementing MOUD in prisons include illicit drug availability, poor understanding of harm reduction and MOUD, stigmatization of MOUD, lack of resources and inconsistency in treatment offerings for prison transfers, and rigid treatment dispensing (Komalasari et al., 2021). Less is known about the environmental, cultural, and structural factors that shape MOUD adoption or expansion in rural jails that often lack the resources needed to implement such methods (Kang-Brown & Subramanian, 2017).

In 2022, the Colorado legislature mandated county jails to provide all three FDA-approved medications by July 1, 2023 (Colorado General Assembly, 2022). While the bill appropriated $3 million to assist county jails in the implementation of MOUD protocols, questions remain concerning the capacity and readiness of rural jails to comply with such a law.

### Current study

This study included interviews aimed at identifying needs, current service availablity, and existing challenges for MOUD in rural jails in Colorado to facilitate the implementation and expansion of MOUD services at these facilities and upon release. The research question was: what barriers to and facilitators of the provision of MOUD services exist for people who are incarcerated in and released from seven rural Colorado jails? Ultimately, this study intended to understand the capacity for these rural jails to implement comprehensive MOUD programs and identify what additional support was needed for success. The study is useful for rural jails of varying sizes, funding streams, and support from sheriffs and staff to provide MOUD. The study results highlight the specific challenges of implementation of the legislative mandate for rural jails.

## Methods

The Biomedical Research Alliance of New York institutional review board exempted this study (study ID# 22-12-356-1183) as minimal risk to participants. Standards for reporting qualitative research were followed (O’Brien et al., 2014).

### Study and setting

For this qualitative study, we investigated the capacity for MOUD service provision in southern Colorado county jails. From March–September 2022, we recruited seven jails operating in nine rural counties of Colorado with varying capacity and participation in the state’s Jail-Based Behavioral Health Services (JBBS) program. Two counties did not have operational jails at the time of the study, while the other counties have jails with potential to engage with a network of community health clinics and hospitals participating in the Colorado MOUD expansion program under Colorado’s Behavioral Health Recovery Act of 2021 (Senate Bills 19 − 001 and 21–137) (Amura et al., 2023). This scale-up program was implemented after the success of the pilot implementation in two Colorado counties with high opioid overdose death rates (Amura, 2022). At the time of the study, the seven jails were not part of the larger MOUD expansion efforts. However, the jails had the potential to engage with the network of community health clinics for provision of continuity of care for patients during incarceration. This study aimed to determine the facilitators of and barriers to these county jails being included in the broader scale-up program in Colorado and their readiness to implement MOUD services. All seven counties participating in this study are designated as rural by the Colorado Department of Local Affairs (Colorado Department of Local Affairs, 2024).

### Recruitment and sources of data

This study includes data from two sources: in-person/virtual jail visits and in-depth interviews. In-person visits were conducted in all but one jail (which included a virtual tour) to assess the facility layout and areas for medical care provision, medication storage, and other physical characteristics that could impact MOUD service delivery. Interview participants (*n* = 19) were recruited via phone or e-mail contact during the spring of 2022, followed by an informational letter about the study. Snowball sampling was used to help identify additional county-specific contacts. In total, 19 people participated in seven interviews conducted at each of the participating county jails. While not designed as a moderated focus group, five of the interviews naturally included multiple participants answering to the same semi-structured questions. Multiple participants were included in single interviews to encourage wider participation and achieve efficiency in use of participants’ time. Face-to-face interviews with two of the researchers on the study team were completed with participants onsite during May–September of 2022. To protect participants’ confidentiality, no demographic data of participants were collected.

Interviews were conducted using a semi-structured interview guide (see Table 1) developed by the research team to assess barriers to and facilitators of providing MOUD. Development of the interview guide was based on concepts found in review of the existing literature. An initial survey was sent to the identified jails in which the information solicited also informed the development of the interview guide. Additionally, the draft interview guide was reviewed by staff from a local federally qualified health center that coordinates MOUD services and was piloted with jail staff from one of the rural counties prior to use in this study for acceptability and appropriateness. Each interview lasted one to two hours. All interviews were audio recorded after verbal consent was obtained. Interviewees were not compensated for their participation.

**Table 1:** Interview Guide

1. Please tell us about how criminal justice-involved individuals move through your facility. a. Where do they come from? Do they often move to other facilities? If released, do they stay in the area or are many of them not residents of this county?
2. Please tell us about how substance use or opioid use is assessed and managed in your facility. a. Screening b. Detoxification c. Service provision
3. What is working well with the existing services or processes related to substance use in your facility?
4. Where do you think there are opportunities to improve services for substance use or opioid use among the inmates in your facility?
5. Can you describe an ideal situation for the inmates here with opioid use or substance use? What is needed for them to get the care they need?
6. What barriers exist to being able to expand services for substance use and opioid use at your facility?
7. Are you familiar with Medication-Assisted Treatment for Opioid Use Disorder? (ex: suboxone, vivitrol, naltrexone, methadone, buprenorphine) a. If no, describe them to interviewee. b. If yes, ask: do you think your facility would be able to implement a medication-assisted treatment program? i. What would be needed to make this successful? ii. What are the biggest challenges you experience in regard to setting up medication-assisted treatment?
8. Do you know if there are health or behavioral/mental health providers in the area that offer substance use or opioid use treatment? MAT specifically? Who else would need to support the idea of MAT expansion in this facility to make it successful?

### Data Analysis

Interview transcripts were created using Otter.ai (Mountain View, CA), reviewed for accuracy, and de-identified prior to upload into an online software tool, Dedoose version 9.0.62 (Los Angeles, CA), for qualitative thematic analysis.

Two team members conducted data coding, engaging in iterative discussions to address alternate interpretations (Elo & Kyngas, 2007; Graneheim & Lundman, 2004; Lincoln & Guba, 1986). To ensure the study’s rigor and trustworthiness, essential elements of qualitative research, the authors gathered information from local experts with knowledge and working experience with SUD or OUD and/or criminal justice, illustrated themes with exemplar quotes, and validated information in subsequent interviews. Field notes were recorded to document additional details and decisions by two researchers immediately after the interviews, ensuring transparency in data collection and to provide additional context during the analysis (Elo & Kyngas, 2007; Lincoln & Guba, 1986; Patton, 2015).

## Results

County or contracted personnel from all seven targeted jails, including sheriffs (*n* = 4), a deputy (*n* = 1), jail nurses (*n* = 4), jail administrators (*n* = 5), and other contracted personnel (*n* = 5), participated in interviews. In six of the seven jails, multiple participants were interviewed at the same time. Of the seven jails, three were actively providing at least one FDA-approved MOUD drug (one gave naltrexone injections and the other two provided both naltrexone and buprenorphine), two had plans to offer or occasionally provided MOUD, whereas the two smaller and least resourced jails did not provide MOUD services. There was also variation in jail bed capacity (8 to 240 beds), nurse staffing (0 to 4.0 full-time equivalents or FTE), medication storage availability, and participation in the state’s JBBS program for continuity of care of incarcerated persons after release (see Table 2). Various themes regarding barriers and facilitators identified by interviewees are summarized below.

**Table 2:** Characteristics of County Jails in Sample (*n* = 7)

County Jail	Bed Capacity	Advocate for Incarcerated Persons	JBBS^a^	Locked Medication Storage	Refrigerated Storage	Nurse	Transition Team^b^	MOUD Types Provided^c^
County A	170	No	Yes	Yes	Yes	1.0 FTE	Yes	B, N, M
County B	82	No	Yes	No	No	No	No	
County C	25	No	No	Unknown^d^	No	No	No	
County D	8	Yes	Yes	Unknown	No	Contracted^e^	Yes	B, N
County E	240	No	Yes	Yes	Yes	4.0 FTE	Yes	B, N
County F	36	Yes	Yes	Yes	Unknown	0.2 FTE	Yes	B, N
County G	70	Yes	No	Yes	No	1.0 FTE	No	N

^a^ Jail-Based Behavioral Services (JBBS) program recipient: https://bha.colorado.gov/behavioral-health/jbbs

^b^ Team or resource dedicated to planning for and connecting individuals to services prior to and during transition/release from jail

^c^ MOUD types provided within jails: buprenorphine (B), naltrexone (N), methadone (M)

^d^ The research team was unable to confirm specific details about medication storage.

^e^ Nurse provided via contracted telehealth services.

*Notes* All data other than JBBS program participation were captured via a pre-interview survey and verified during interviews. County A reported providing methadone only once previously; Counties D, F, and G reported the ability to offer MOUD if needed but had not had any recent or active prescriptions at time of interview

### Facilitators of MOUD

#### Support for MOUD

Most sheriffs and jail staff interviewed were supportive of offering MOUD and related services. Two of the sheriffs were already aware of the upcoming legislation and asked questions on how to get prepared. Despite others not being aware of the legislation when asked if they would be willing to offer MOUD in the jails, they were willing to consider it with appropriate support and resources in place. All jail nurses were in support of offering or increasing MOUD services. They understood the appropriate use of medication as treatment for people who are incarcerated. One stated, “whether it’s a Vivitrol or Suboxone, [it] is not just giving up free drugs. It’s gonna help my community” (Interview 5). One jail staff noted positive changes in the jail since offering MOUD: “We absolutely see the benefits. When staff assaults go down, when inmate assaults go down. It’s measurable and you have to tie it to something. It’s not just because we’re doing a great job managing them” (Interview 5).

#### Collaborative partnerships

Some jails provided behavioral health services through collaborative partnerships with other entities in the area that support their programs. One jail staff said, “I’ve had a call where I’ve requested somebody from behavioral health to speak with me and they’ve come all the way from [another county], which is almost a two-hour drive” (Interview 3). Three jails that offered MOUD had existing relationships with Colorado’s JBBS program and routinely administered MOUD during incarceration or prior to release.

#### Jail nurses

The jails that had existing MOUD programs had nurses on staff. The research team observed differences in programs that had nurses in their jails compared to those that did not. The jails with nurses had withdrawal protocols and standing orders for medications to treat symptoms; made attempts to work on transition plans and often to connect people who are incarcerated with services upon release, which was more likely to happen when nurses were aware of the plans for release and on duty; and dedicated space for medication storage and processes to account for medications. Other jail staff were trained when needed to provide medications. One nurse also mentioned commitment and ability to meet the needs of people who are incarcerated through education, counseling, transition planning, anticipatory guidance or other means: “That’s what’s important about when you do build the rapport. I kind of can feel them out, know what position they’re in this day, but it is an opportunity that provides for them the resources they need” (Interview 4).

### Barriers to MOUD

#### Physical space limitations

Space limitations in the smaller jails impeded the ability to give comprehensive care. The smaller jails lacked dedicated or secure space for storage of medications. In addition, the older jails often did not have rooms for medical exams, treatment, or observation of people who are newly incarcerated and may experience withdrawal symptoms. One interviewee said, “The problem with that is it’s not necessarily a private setting. So, to do any sort of treatment or anything like that would be problematic” (Interview 6). The research team confirmed these findings during in-person visits to the facilities. Much of this care took place in the booking area or inside the cells. Spaces often were used for multiple purposes. Additional treatment offerings like group therapy or peer support were even harder to facilitate with the space constraints in the smaller jails. One interviewee said,

It’s the space, you know, if I had a media room where I could offer, you know, groups every day that the guys could participate in as far as you know, recovery, or men’s groups or whatever…. I just, I have no way to do that” (Interview 7)

#### Distance to services

The long distances between the jails and established health services in some counties made it challenging to get these services for people while incarcerated, especially if telehealth was not available. One interviewee mentioned,

Mental health can be anywhere from another two to four hours, depending on how far…it’s very different out here compared to the city. I mean, you know, Pueblo, Colorado Springs, they have all those resources there at the hospitals. We don’t (Interview 1)

The reference to “mental health” services refers to the partnerships in rural communities with therapists or licensed providers that support MOUD treatment and services, interviewees would often use the term to describe services for OUD/SUD. The long distance to services also posed challenges for transition planning and connecting people with services upon release.

#### Lack of providers in the area

Several jail staff noted it is hard to find local nurses who are available to work in the jails. Some counties do not have specialists like psychiatrists or providers who can prescribe MOUD. One interviewee said, “The shortage of medical providers is horrendous in this area. This part of the state hasn’t had a psychiatrist in the area in at least 12 years” (Interview 6). As a result, jail staff and sheriffs were forced to learn skills to address the needs of people who are incarcerated. “The American Sheriff is the number one provider of mental health services in America” (Interview 6), said one interviewee.

#### Staffing and training issues

One of the most common barriers discussed was staffing issues. These jails are often unable to retain employees in their facilities and patrol officers to adequately cover their county and/or staff their jail. “We are extremely understaffed right now” (Interview 7), said one jail staff. One jail administrator said the jail has not had a cook for months to make food for people in the jail, so the sheriff’s family members came in and helped prepare food. Staff in rural jails cover multiple roles, including giving medications and coordinating visits with treatment providers. If staff must cover duties like cooking meals or even finding someone else to cook meals, it puts undue burden on the already strapped resources in these facilities and pulls them away from the provision of services they typically manage. Other jail administrators could not find nurses to work even when they had the resources to pay for those positions.

As a result of the staffing challenges, the jails struggled with high turnover and inexperienced staff. “County jails, especially in the small rural communities are really in trouble…. I think I’ve been here eight years and the captain said we’ve been through 150 employees in eight years” (Interview 4), said one interviewee. Turnover complicated training, which prevented the maintenance of expertise and put safety at risk. Some people who are incarcerated were relocated to other jails due to staffing issues. One sheriff mentioned that having new staff often makes change easier to implement, but they lack the consistency in knowledge and expertise to ensure programs like MOUD remain successful: “[My staffing situation] is a double-edged sword; I have a ton of young staff, zero experience. So, they are more acceptable of change. The flip side is they don’t know how to deal with inmates yet” (Interview 5). Other staff mentioned having ample young staff but with little experience which made provision of MOUD and related services harder. Jail administrators talked about the need for staff training to introduce or expand MOUD services: “The first thing we would need is we have to have, you know, personnel that’s trained” (Interview 3).

#### Inadequate funding

Barriers were magnified by limited or restricted budgets without the flexibility to pay for needed services. Some smaller jails did not have enough funds to pay for a nurse or contract with health providers. This was a heightened issue for jails not currently involved with JBBS, which provides additional funding and support for mental health and substance use services in jails. One interviewee mentioned, “Honestly, I think the jail is paying for a lot of it. JBBS will cover inmates if they qualify for the program, sometimes the jail has to eat the cost” (Interview 4).

Further, people who were covered by Medicaid lost their insurance coverage upon incarceration and thus medications and services had to be funded through the jail budget, JBBS (if available), or other funding sources. Also, it was unclear who should pay for medications as some were expensive (naltrexone injections run around $1,000 each). This continued to be a barrier when people were released and did not have active insurance coverage to pay for transition services and MOUD.

#### Perceived risk of diversion

In most jails examined there was a list of unapproved medications that cannot be administered or brought into the building. These lists included substances with a high risk of potential for misuse and/or addiction and almost all included MOUD (namely methadone and buprenorphine). Due to the inconsistency in regulations, in most jails there was no standard for approving MOUD use. Furthermore, multiple interviewees noted the diversion of medications that might be misused, despite having processes in place to prevent or mitigate diversion. One interviewee mentioned, “Diversion’s huge. We know it happens…because they’re finding little stockpiles on shakedowns” (Interview 5). Another said, “Our problem is sometimes you have to watch them because they’ll lip it or something, and they start sharing them in the unit with other people….” (Interview 1).

#### Lack of support for MOUD

The use of MOUD was a new topic of discussion for some of the jail staff. Interviewees noted that there is a lack of acknowledgment of how prevalent SUD was in jails among the general public and some local officials. One interviewee said, “We have to work with the commissioners, like I said, they aren’t here every day, like we are, where we see these needs. It’s kind of out of sight out of mind” (Interview 7).

Several interviewees also seemed hesitant due to not having good information about the clinical evidence of MOUD benefits and what the path to recovery entails. Some jail staff stated they have not seen success with MOUD for people who are incarcerated: “I have yet to see really any efficacy in any of it for this population” (Interview 2).

#### Recidivism and lack of transition to post-release services

Interviewees noted that many individuals who get released from jail did not have the necessary support to be successful with recovery (e.g. lack of transportation, no relationship with a treatment provider, no source of income to cover expenses). One interviewee said,

The day they walk out, all the papers are done, we turn it in, but I think it takes a little bit of time. And unfortunately, that little bit of time can be the difference between somebody following through and somebody going out and reusing (Interview 8)

Also, not all jails had transition programs to help connect individuals with services to provide continuity of care. This is confounded with Medicaid being terminated upon incarceration and the lag time to reinstate services/benefits after release. Many jail staff noted there is a high rate of recidivism because of offenses related to substance use. One mentioned, “I think the system is failing these guys to bring them in a detention facility for what, three months? Then they’re out doing the same thing all over again.” (Interview 1).

## Discussion

This study examined the capacity for MOUD service provision in seven rural Colorado county jails via interviews with employed and contracted jail personnel. While facilitators of MOUD adoption or expansion were identified, far more barriers were found including physical space limitations, distance to services, lack of providers in the area, staffing turnover and training issues, inadequate funding, and perceived risk of diversion, which is consistent with previous findings (Brezel et al., 2020). However, most interviewees reported that they were willing to adopt or increase MOUD services, which could reflect the acknowledgment of the current state-level mandate.

Current MOUD provision within the jails enrolled in this study was limited to naltrexone and/or buprenorphine. These MOUD modalities present unique challenges such as cost, training for staff to administer, timing and planning, secure medication storage, and perceived risk of diversion from staff. Methadone induction in jails has been shown to decrease non-fatal overdoses post-release and increase participation in treatment after release (Brinkley-Rubinstein et al., 2018). However, methadone administration likely poses greater challenges for rural jails including prescribing and dispensing restrictions and lack of local licensed opioid treatment providers or mobile methadone treatment options (Rising et al., 2022). All jails in this study at the time of interviews had approved medication lists for their jails and MOUD were often listed on the “restricted” lists. This will have to change to make MOUD provision feasible for those jails not already offering MOUD.

Policy implementation depends on service delivery by frontline workers (Stohr & Zupan, 1992), such as jail nurses and staff, who ensure people who are incarcerated with OUD receive: (1) MOUD, usually in conjunction with therapy or counseling services and peer support; (2) reasonable withdrawal treatment and best-practice medical intervention to manage OUD/SUD symptoms; and (3) transition services and post-release resources, including a prescription for MOUD, coordination of care with a substance use provider, and a supply of naloxone. Rhode Island was the first state to implement a comprehensive program for OUD in a correctional system in 2016 and had statewide decreases in overdose deaths and post-incarceration overdose deaths after the program was in place (Clarke et al., 2018). Following the lessons learned from Rhode Island (Brinkley-Rubinstein, 2019), Massachusetts (Evans et al., 2021), and Maryland jails (Belcher et al., 2021) other states could identify ways to implement substance use screening, counseling and treatment with MOUD during incarceration, and coordination with MOUD providers post-release. In addition, lessons learned from other state’s jail programs can help with overcoming barriers like the perceived risk of diversion. A study of the program in Massachusetts demonstrated that the perceptions surrounding buprenorphine diversion were more infrequent than expected and diversion was preventable (Evans et al., 2022a, b). Proactive strategies implemented in the jails helped change perceptions about the risk for diversion and included routine but flexible dosing protocols, education and monitoring, patient involvement in assessing reasons for diversion, and written policies to adjudicate diversion consequences (Evans et al., 2022a, b).

In rural areas, additional barriers exist to provide MOUD to people who are incarcerated due in part to geographical distance to services (Mitchell et al., 2022) and lack of treatment providers (Bridges et al., 2023). Funding was also a barrier to providing MOUD identified in this study, as the rural jails were under-resourced and faced challenges of maintaining adequate staffing. In addition, new staff without experience could introduce safety risks but empirical evidence for this is mixed (Ellison & Gainey, 2020). Many rural facilities may be inadequate to provide MOUD and county jail officials have to decide how to spend their limited budgets.

## Limitations

The findings of this study are limited to only a small number of rural county jails in Colorado. Given their geographical proximity, these jails may have more in common with one another than with rural jails in other areas of Colorado or in other states. Also, the perspectives of study participants do not include those of people who are incarcerated or other community stakeholders such as substance use treatment providers or county commissioners. The timing of the study may have played a role in the willingness of some county jails to begin preparing for MOUD expansion considering the 2023 statewide mandate. Finally, the fact that some interviews included multiple participants may have impacted participant’s willingness to respond to questions where their views may have been seen as negative or opposite of what other participants views were. Despite using the group interview strategy for convenience of the participants, it could have impacted what content was shared during the interviews.

## Conclusions

This study found that while rural jails have the ability to facilitate MOUD in their facilities, numerous barriers that jail administrators often have less control over remain such as funding for medications, distance to health services, and the availability of trained staff including nurses and other health professionals, which currently limit the ability of rural Colorado jails to achieve a healthier and more supportive correctional environment. These findings are timely in light of recent state legislative efforts, such as in Colorado, that require availability of MOUD for people who are incarcerated. The findings of this study can be useful for rural jails of varying sizes, funding streams, and levels of support from sheriffs to provide MOUD. These findings illustrate specific challenges in small rural jails and highlight the burden of an unfunded mandate. This could be used as an opportunity for other states to understand how to implement mandates (funded or unfunded). Future research determining how smaller rural jails can better meet the needs of populations of people who are incarcerated with OUD, and specifically how methadone treatment can be successfully implemented in rural jails, should be prioritized.

## Data Availability

Data supporting the findings of this study are held by The Schreiber Research Group and are not publicly available due to privacy restrictions.
